# Development of a Noninvasive Prediction Score for Post-Capillary Pulmonary Hypertension in a Multi-Ethnic Asian Population

**DOI:** 10.1016/j.jacasi.2026.03.012

**Published:** 2026-05-05

**Authors:** Haowen Jiang, Stephanie Loh, Shaw Yang Chia, Wen Ruan, Jin Shing Hong, Aidila Binte Ismail, Chee Lan Lim, Michelle Koh, Duu Wen Sewa, Ghee Chee Phua, Ying Zi Oh, Sue-Ann Ng, Cassandra Hong, Andrea Low, Matthew Seow, Soo Teik Lim, Ju Le Tan, Jonathan Yap

**Affiliations:** a National Heart Centre Singapore, Singapore; b Duke-NUS Medical School, Singapore; c Singapore General Hospital, Singapore; d Changi General Hospital, Singapore

**Keywords:** heart failure, pulmonary hypertension, right heart catheterization

## Abstract

**Background:**

Noninvasive prediction of postcapillary pulmonary hypertension (PH) is a useful clinical tool. However, existing scores are not well evaluated in Asian populations.

**Objectives:**

This study aimed to develop a novel prediction score for postcapillary PH in an Asian cohort.

**Methods:**

A single-center retrospective cohort study was performed in patients who had undergone right heart catheterization from July 2018 to June 2022. Patients with an ejection fraction <50% or moderate or worse left valvular disease were excluded. Postcapillary PH was diagnosed with a mean pulmonary arterial pressure >20 mm Hg and pulmonary capillary wedge pressure (PCWP) >15 mm Hg. A novel logistic regression model (POISE [Prediction Of raISEd wedge pressures]) was developed, with validation by bootstrapping. Comparisons against the OPTICS and H2FPEF scores were performed. Sensitivity analysis was performed excluding patients with combined pre- and post-PH.

**Results:**

A total of 266 patients were included, of whom 110 had a raised PCWP >15 mm Hg. Body mass index >30 (adjusted OR: 2.79; 95% CI: 1.42-5.47), atrial fibrillation (adjusted OR: 2.26; 95% CI: 1.01-5.02), E/e’ ratio (adjusted OR: 1.058, 95% CI: 1.01-1.10), and left atrial index >34 cm^2^/m^2^ (adjusted OR: 2.07; 95% CI: 1.13-3.78) were significant predictors of raised PCWP (C-statistic: 0.72; 95% CI: 0.65-0.78). At a chosen cutoff of ≥0.72, POISE had a specificity of 97.4% (95% CI: 95.0%-99.9%) and sensitivity of 10% (95% CI: 4.4%-15.6%). Internal validation and sensitivity analysis excluding patients with combined pre- and post-PH revealed C-statistics of 0.70 (95% CI: 0.70-0.79) and 0.75 (95% CI: 0.67-0.81), respectively, outperforming both OPTICS and H2FPEF scores.

**Conclusions:**

The high specificity and better performance (vis à vis existing scores) of POISE may help in identifying a subset of Asian patients with postcapillary PH, reducing the need of right heart catheterization. POISE is available as an online risk calculator.

Postcapillary pulmonary hypertension (PH) is a commonly encountered clinical condition characterized by increased venous pressure in the pulmonary circulation due to underlying left heart disease and is estimated to affect 1% of the patient population.^1^ Because of differences in prognosis and treatment of pre- and postcapillary PH, it is crucial to differentiate the 2 groups.^1^ The treatment of postcapillary PH involves management of the underlying left heart disease and fluid status. On the other hand, precapillary PH, which includes pulmonary arterial hypertension (PAH) as well as chronic thromboembolic PH,^1^ requires specific treatment modalities. PAH-specific treatment is unlikely to be beneficial in postcapillary PH and may even be harmful.^2^

Currently, right heart catheterization (RHC) remains the definitive method to differentiate the 2 groups. However, RHC is invasive, costly, and may not be readily accessible in developing areas because of lack of expertise and resources.^3^ In addition, patients especially in Asia, may decline undergoing an invasive investigation due to cultural/social reasons. Without an RHC, the differentiation of pre- and postcapillary PH can be challenging in the absence of overt left heart disease,^4^ especially with a shifting demographic of patients with PAH who tend to be older with more comorbidities and hence a greater probability of postcapillary PH.^5^ The OPTICS and H2FPEF scores have been suggested to be useful in predicting postcapillary PH in this context and have been previously validated in European cohorts.^4^^,^^6^ However, distinct differences exist between Asian and Western populations^7^ and hence the utility of the scores in an Asian population is unknown. We thus aim to develop and validate a novel risk prediction score for postcapillary PH in an Asian population and compare it to the performance of existing risk scores.

## Methods

We report our study in accordance with the TRIPOD statement.^8^

### Study population

Singapore has a multiethnic population of 4.18 million, comprising 74% Chinese, 13% Malay, and 9% Indian.^9^ This is a retrospective study at a single tertiary cardiac center in Singapore from July 2018 to June 2022. All patients who had undergone successful RHC with measurement of pulmonary capillary wedge pressures (PCWP) were included regardless of the final diagnosis. Patients with left ventricular ejection fraction (LVEF) of <50%, left-sided valvular heart disease grade moderate or worse, or any other significant left heart pathology that may account for postcapillary PH were excluded. If patients had undergone more than 1 RHC, data from the first RHC was used. An ethical waiver was obtained from the institution’s institutional review board as the study involved the use of deidentified data (CIRB: 2025/1108). Adult participant consent was not required because this was a retrospective cohort review.

### Study procedure

Demographics, clinical characteristics, and investigations were collected from the electronic medical records. Potential variables studied include age, obesity (defined as body mass index >30 kg/m^2^), a medical history of hypertension, diabetes (fasting blood glucose ≥7 mmol/L (126 mg/dL), or taking oral hypoglycemic agents or insulin), dyslipidemia (fasting total cholesterol >5.2 mmol/L [200 mg/dL]), previous left-sided valvular surgery (without residual left valvular heart disease), atrial fibrillation (AF) (paroxysmal or persistent), smoking history >1 pack year. From the electrocardiogram (ECG), SV_1_ (S deflection in V_1_ in millimeters) and RV_6_ (R deflection in V_6_ in millimeters) were collected as markers of left ventricular hypertrophy. From the transthoracic echocardiogram, left atrial (LA) dilatation (LA index >34 mL/m^2^), ratio of peak early mitral inflow velocity to peak early diastolic velocity of the septal annulus (E/e′), LVEF, and estimated pulmonary artery systolic pressure (PASP) were collected.

### Outcome measures

The primary outcome was diagnosis of postcapillary PH, defined on RHC as a mean pulmonary artery pressure of >20 mm Hg and PCWP >15 mm Hg. PCWP was measured at end-expiration at rest and over multiple breathing cycles. A separate sensitivity analysis also was performed with exclusion of patients with combined pre- and postcapillary pulmonary hypertension (CpcPH), defined as PCWP >15 mm Hg with pulmonary vascular resistance (PVR) >2 WU.^1^

### Statistical analysis

Patient characteristics are presented as mean ± SD for continuous variables and absolute number (percentage) for categorical values. Unpaired Student's *t*-tests and chi-square tests were performed to compare patient characteristics between the 2 groups. A *P* value of <0.05 was taken as statistically significant. Randomness of missing data was evaluated via Little’s missing completely at random (MCAR) test.^10^ If the MCAR test was significant (*P* < 0.05), sensitivity analysis was performed by comparing covariates of included with excluded cases and including additional significant covariates into the prediction model. Statistical analysis was performed using RStudio Version 4.1.2 and IBM SPSS version 25.0 (IBM Corp).

#### Model development

We developed the POISE (Prediction Of raISEd wedge pressure) risk score for postcapillary PH in Asian individuals. Independent Student's *t*-tests and chi-square tests were used to determine variables associated with a raised PCWP. ORs and their 95% CIs were calculated. Missing data were handled via complete case analysis. Univariate and multivariate logistic regression analyses were performed to determine the final independent predictors of a raised PCWP, with variables with a *P* value of <0.05 selected.

Values predicted by POISE belong to a range from 0 to 1 and represent the probability of raised PCWP. The model is as follows:

$$p=\frac{1}{1+e^{-y}}$$

where *p* is the predicted probability of raised PCWP, *e* is a base of natural logarithm, and *y* is a linear combination of variables (*x*_*i*_) and their estimators (*b*_*i*_) included in the model:

$$y=b_{0}+b_{1}x_{1}+b_{2}x_{2}+\ldots +b_{n}x_{n}$$

For binary predictors (eg, obesity status), *x*_*i*_ = 1 if present, and 0 if absent.

A cutoff value was chosen with a target specificity of >97% based on prior publications,^4^^,^^6^ with a view to minimize the risk of misdiagnosis of patients with precapillary PH. Hence, the Youden index (which maximizes accuracy) was not used in the determination of the cutoff.

#### Model discrimination, validation, and calibration

Model discrimination was measured by the area under the curve (AUC) and its 95% CI. Model sensitivity, specificity, positive predictive value (PPV), negative predictive value (NPV), and accuracy were also calculated as metrics of model performance. When evaluating an association within any given data set, the apparent strength of that association may be overestimated because of idiosyncrasies of the data. To correct for this overoptimism (so-called internal validation), bootstrapping^11^ was used as a resampling technique. Here, 133 (50%) new data sets were used by randomly sampling selected subjects from the main data set, with replacement. Next, stepwise multivariable logistic regression analysis was performed on each of these 133 data sets, by considering the models with significant univariable *P* values (*P* < 0.05). Internal bootstrap validation (133 bootstrap samples) was used to provide optimism-corrected estimates. It was applied to each of the imputed data sets. The optimism is the decrease in model performance between the bootstrap and the original samples, which can adjust the developed model for overfitting. The corrected calibration slope was used as a shrinkage factor for the regression coefficients, and AUC with 95% CI corrected for overoptimism was estimated.

Model calibration (the agreement between predicted and observed outcomes) was expressed graphically, with observed risks plotted on the y-axis against predicted risks on the x-axis. The corresponding calibration intercept and slope were calculated. The calibration slope evaluates the spread of the estimated risks and has a target value of 1. The calibration intercept is an assessment of calibration-in-the-large and has a target value of 0. Perfect calibration shows predictions lying on the 45° line of the calibration plot (ie, a slope of 1 and intercept of 0).

#### External validation of existing risk scores

The OPTICS score includes 7 noninvasive variables including ECG and transthoracic echocardiogram values to predict the likelihood of postcapillary PH. In summary, the variables are obesity, diabetes mellitus, AF, dyslipidemia, valvular surgery, ECG changes, and LA dilatation. At a cutoff of ≥104, the score had an AUC of 0.809-0.826, specificity of 96%-100%, and sensitivity of 22%-28% on external validation in European cohorts.^4^^,^^6^

The H2FPEF score was originally developed for the prediction of heart failure with preserved ejection fraction, but because of similarities in pathophysiology and risk factors between heart failure with preserved ejection fraction and group 2 PH, it has been suggested to be able to predict a raised PCWP as well. It consists of 6 variables: obesity, hypertension, AF, PH, elderly, and filling pressures on echocardiography. At a cutoff of ≥6, the H2FPEF score had an AUC of 0.792-0.859, specificity of 91%-92%, and sensitivity of 48%-70% in European cohorts.^4^^,^^6^

A summary of both these existing risk scores can be found in Supplemental Tables 1 and 2.

The established cutoffs for the OPTICS and H2FPEF scores of ≥6 and ≥104 were applied retrospectively to predict the presence of raised PCWP (defined as >15 mm Hg) and the results compared with the actual PCWP from RHC. Model performance was evaluated via sensitivity, specificity, PPV, and NPV at the prespecified cutoffs, and via AUC and compared with the POISE score. Decision curve analysis was performed to determine the net benefit at 25% and 30% thresholds by weighing the harm of false positives against the benefit of true positives.^12^

## Results

### Patient characteristics

A total of 266 patients were finally included (mean age = 61.5 years, 101 [38.0%] men, 182 [68.4%] Chinese) (Supplemental Figure 1). A total of 110 of 266 patients (41.4%) had a raised PCWP >15 mm Hg of whom 68 of 110 (25.6%) had CpcPH with both PCWP >15 mm Hg with PVR >2 WU. When compared with patients with a normal PCWP, patients with a raised PCWP were more likely to be obese, with a higher incidence of cardiovascular comorbidities, AF, and LA dilatation (Table 1).

**Table 1:** Clinical Characteristics of the Study Population

	Overall (N = 266)	PAWP ≤15 (n = 156)	PAWP >15 (n = 110)	*P* Value
Demographics				
Age, y	64.0 (53.0, 71.6)	64.1 (54.3, 70.7)	63.8 (51.9, 72.5)	0.995
Male	101 (38.0)	55 (35.3)	46 (41.8)	0.277
Ethnicity				0.703
Chinese	182 (68.4)	110 (70.5)	72 (65.5)	
Malay	44 (16.5)	25 (16.0)	19 (17.3)	
Indian	23 (8.6)	11 (7.1)	12 (10.9)	
Others	17 (6.4)	10 (6.4)	7 (6.4)	
Height, m	1.57 (1.52, 1.66)	1.57 (1.51, 1.66)	1.57 (1.52, 1.66)	0.299
Weight, kg	60.6 (51.0, 72.1)	58.8 (50.0, 69.8)	63.6 (53.5, 75.1)	**0.004**
Body mass index >30 kg/m^2^	49 (18.4)	22 (14.1)	27 (24.5)	**0.030**
Body mass index, kg/m^2^	24.0 (21.0, 28.4)	23.4 (20.5, 27.6)	25.0 (21.4, 29.8)	**0.014**
Clinical characteristics				
Diabetes mellitus	69 (25.9)	34 (21.8)	35 (31.8)	0.066
Hypertension	140 (52.6)	75 (48.1)	65 (59.1)	0.076
Dyslipidemia	128 (48.1)	75 (48.1)	53 (48.2)	0.987
Smoker	29 (10.9)	21 (13.5)	8 (7.3)	0.111
Ischemic heart disease	59 (22.2)	33 (21.2)	26 (23.6)	0.631
Previous PCI	22 (8.3)	11 (7.1)	11 (10.0)	0.390
Previous CABG	6 (2.3)	3 (1.9)	3 (2.7)	0.664
Valve disease/Previous valve surgery	17 (6.4)	7 (4.5)	10 (9.1)	0.131
Atrial fibrillation	38 (14.3)	13 (8.3)	25 (22.7)	**0.001**
Echocardiography				
Left atrial volume index, mL/m^2^	30.1 (22.2, 42.1)	27.9 (21.1, 35.7)	36.1 (24.9, 51.5)	**<0.001**
Left atrial volume index >34 cm^2^/m^2^	106 (39.8)	46 (29.5)	60 (54.5)	**<0.001**
E/e’ ratio	10.0 (7.9, 14.1)	9.4 (7.3, 13.0)	11.6 (8.6, 18.1)	**<0.001**
Pulmonary artery systolic pressure, mm Hg	52.0 (39.0, 65.0)	50.0 (36.0, 69.0)	52.0 (40.0, 62.0)	0.648
Left ventricular ejection fraction, %	60.0 (56.0, 64.0)	60.0 (57.0, 64.0)	60.0 (55.0, 64.3)	0.250
Laboratory results				
NT-proBNP, pg/mL	982.5 (221.8, 2431.3)	736.0 (180.5, 2137.5)	1253.0 (304.0, 3552.0)	0.123
Hemoglobin, g/dL	12.4 (10.9, 13.9)	12.8 (11.5, 14.0)	11.7 (9.9, 13.8)	**0.003**
Platelet count, × 10^3^/μL	221.5 (180.5, 293.3)	242.0 (192.8, 304.8)	203.5 (156.0, 255.3)	**0.001**
Creatinine, μmol/L	76.0 (57.0, 102.0)	71.0 (56.0, 90.0)	85.5 (60.3, 132.5)	**0.002**
Sodium, mmol/L	139.0 (137.0, 141.0)	139.0 (137.0, 141.0)	139.0 (136.0, 141.0)	**0.045**
Potassium, mmol/L	4.2 (3.9, 4.5)	4.2 (4.0, 4.5)	4.2 (3.9, 4.5)	0.460
Right heart catheterization				
Heart rate	74.0 (65.8, 85.0)	73.0 (66.0, 82.0)	75.0 (64.5, 87.5)	0.242
Mean right atrial pressure	9.0 (5.5, 14.0)	6.0 (4.0, 9.0)	14.0 (9.0, 19.0)	**<0.001**
Mean pulmonary artery pressure	33.0 (23.0, 44.0)	28.0 (20.0, 40.0)	36.5 (29.8, 48.3)	**<0.001**
Right ventricular systolic pressure	51.0 (37.0, 70.3)	46.0 (33.0, 67.3)	57.5 (45.0, 74.8)	**<0.001**
Left ventricular systolic pressure	135.0 (120.0, 154.0)	133.0 (118.0, 150.0)	135.5 (122.5, 159.0)	0.112
Aortic systolic blood pressure	130.0 (112.3, 152.8)	128.5 (111.8, 152.3)	133.0 (113.0, 153.0)	0.432
Aortic diastolic blood pressure	70.0 (62.3, 81.0)	70.0 (62.0, 80.3)	71.0 (63.0, 81.3)	0.736
Cardiac output, L/min	4.8 (3.8, 5.9)	4.5 (3.7, 5.5)	5.1 (4.0, 6.6)	**0.003**
Pulmonary vascular resistance, WU	3.1 (1.9, 6.1)	3.5 (2.1, 6.9)	2.8 (1.7, 5.2)	0.066

Values are median (IQR) or n (%). **Bold** values represent a *P* <0.05.

CABG = coronary artery bypass graft; NT-proBNP = N-terminal pro–B-type natriuretic peptide; PAWP = pulmonary artery wedge pressure.

### Predictors of postcapillary PH

Among 11 variables evaluated on univariate analysis, 4 variables were significant and remained significant on multivariate analysis as predictors of postcapillary PH. These included obesity (adjusted OR [adj-OR]: 2.79; 95% CI: 1.42-5.47; *P* = 0.003), presence of AF (adj-OR: 2.26; 95% CI: 1.01-5.02; *P* = 0.047), E/e’ ratio (adj-OR: 1.058; 95% CI: 1.01-1.10; *P* = 0.021), and LA index >34 cm^2^/m^2^ (adj-OR: 2.07; 95% CI: 1.13-3.78; *P* = 0.018). See Table 2. The multivariate analysis was conducted on a complete dataset (N = 266) with no missing values among the variables of interest, avoiding the need for imputation or listwise deletion.

**Table 2:** Univariate and Multivariate Analysis of Potential Parameters for Inclusion Into the Novel Risk Score

	Univariate *B* (95% CI)	Univariate OR (95% CI)	*P* Value	Multivariate *B* (95% CI)	Multivariate OR (95% CI)	*P* Value
Intercept				−1.642 (−2.268 to −1.017)		**<0.001**
Age >60, y	−0.048 (−0.546 to 0.449)	0.953 (0.579-1.567)	0.849			
Body mass index >30 kg/m^2^	0.684 (0.058-1.310)	1.981 (1.060-3.705)	**0.032**	1.027 (0.354-1.700)	2.792 (1.424-5.474)	**0.003**
Smoker	−0.685 (−1.539 to 0.169)	0.504 (0.215-1.184)	0.116			
Diabetes	0.516 (−0.037 to 1.068)	1.675 (0.964-2.910)	0.068			
Hypertension	0.445 (−0.048 to 0.938)	1.560 (0.953-2.554)	0.077			
Atrial fibrillation	1.174 (0.452-1.896)	3.235 (1.572-6.660)	**0.001**	0.813 (0.012-1.614)	2.255 (1.012-5.023)	**0.047**
SV1 + RV6 on ECG	0.012 (−0.017 to 0.042)	1.012 (0.983-1.043)	0.417			
Left atrial volume index >34 cm^2^/m^2^	1.054 (0.545-1.563)	2.870 (1.725-4.775)	**<0.001**	0.727 (0.126-1.329)	2.069 (1.134-3.776)	**0.018**
E/e’ ratio	0.087 (0.041-0.133)	1.091 (1.042-1.142)	**<0.001**	0.056 (0.008-0.104)	1.058 (1.008-1.110)	**0.021**
Left ventricular ejection fraction	−0.027 (−0.074 to 0.019)	0.973 (0.928-1.020)	0.250			
Pulmonary artery systolic pressure	0.003 (−0.010 to 0.015)	1.003 (0.991-1.015)	0.646			

**Bold** values represent a *P* <0.05.

ECG = electrocardiogram; RV_6_ = R deflection in V_6_ in millimeters; SV_1_ = S deflection in V_1_ in millimeters.

The final equation of POISE for prediction of postcapillary PH is as follows:

$$\log\left(\frac{p}{1-p}\right)=-1.642+1.027\cdot Obesity+0.727\cdot \;LA\;dilation+0.056\cdot E/e^{\prime }Ratio+0.813\cdot AF$$

POISE is available as an online probability calculator^13^ (Supplemental Figure 2).

### Performance of risk scores and comparison with existing risk scores

**Figure 1:**
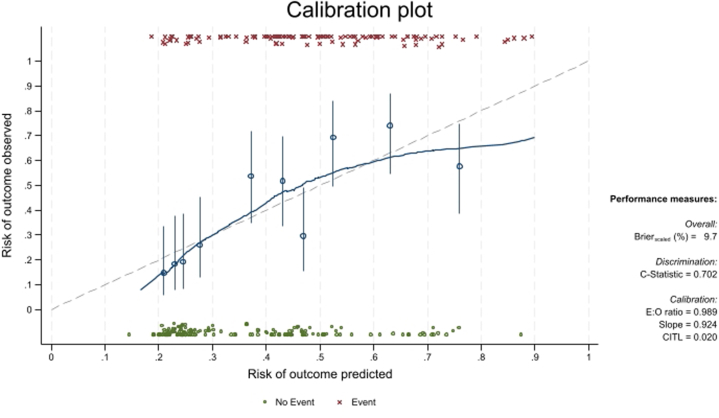
Calibration Plot of POISE Score in the Overall Cohort Calibration plot depicting line of best fit of the POISE (Prediction Of raISEd wedge pressures) score in the overall cohort via internal validation via bootstrapping demonstrating a good discrimination value of 702 (0.643-0.762).

**Figure 2:**
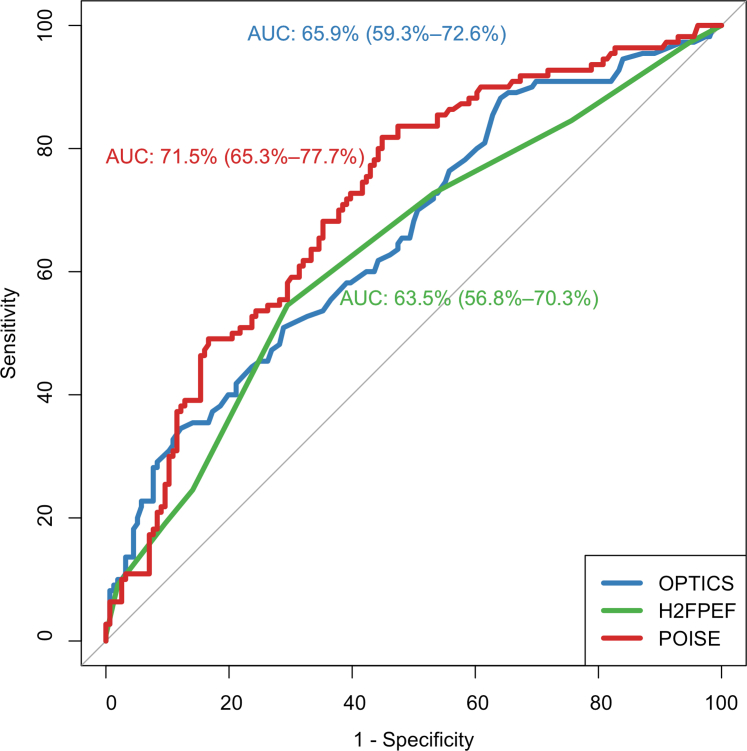
Receiver Operator Curves of POISE, OPTICS, and H2FPEF Receiver operator curve and corresponding area under the curve (AUC) of the novel risk prediction score (POISE) in red, with comparison with the OPTICS score (blue) and H2FPEF score (green), demonstrating a higher AUC for the novel risk prediction score in an Asian cohort. Abbreviation as in Figure 1.

In the overall cohort, POISE demonstrated a C-statistic of 0.715 (95% CI: 0.653-0.777). Validation by internal bootstrapping (n = 133) demonstrated a good discriminative ability for POISE with a C-statistic of 0.702 (95% CI: 0.643-0.762) with a good fit on calibration plot (Figure 1). This was higher than the AUCs of the OPTICS and H2FPEF scores in our local population, with AUCs of 0.659 (95% CI: 0.593-0.726) and 0.635 (95% CI: 0.568-0.703), respectively (Figure 2). Sensitivity performed excluding patients with CpcPH (n = 68) also demonstrated superior C-statistic of POISE (0.728; 95% CI: 0.657-0.798) against both the OPTICS (0.690; 95% CI: 0.607-0.772) and H2FPEF scores (0.608; 95% CI: 0.515-0.701) (Supplemental Figure 3). Decision curve analysis suggests a higher net benefit of the POISE score at both the 25% (POISE: 0.251; 95% CI: 0.180-0.321; OPTICS: 0.227; 95% CI: 0.153-0.301; H2FPEF: 0.198; 95% CI: 0.120-0.277) and 30% thresholds (POISE: 0.224; 95% CI: 0.154-0.294; OPTICS: 0.218; 95% CI: 0.149-0.286; H2FPEF: 0.169; 95% CI: 0.092-0.247) (Supplemental Table 3). On internal validation of POISE excluding patients with CpcPH, the C-statistic was 0.692 although this is limited by the smaller sample size and large confidence interval (95% CI: 0.572-0.796) (Supplemental Figure 4).

At a chosen prespecified cutoff of ≥0.720, POISE had a specificity of 97.4%, sensitivity of 10.0%, PPV of 73.3%, and NPV of 60.6%. In comparison, OPITCS had a specificity of 96.2%, sensitivity of 8.2%, PPV of 60.0%, and NPV of 59.8%; H2FPEF had a specificity of 90.4%, sensitivity of 19.1%, PPV of 58.3%, and NPV of 61.3% in the present cohort. Exclusion of patients with CpcPH demonstrated fairly similar results (Table 3).

**Table 3:** Sensitivity, Specificity, PPVs, and NPVs of POISE, OPTICS, and H2FPEF Scores

	Overall Cohort (N = 266)	Excluding Patients With CpcPH (n = 198)
POISE		
Sensitivity	10 (4.4-15.6)	14.5 (6.7-24.5)
Specificity	97.4 (95.0-99.0)	98.0 (95.3-100)
PPV	73.3 (51.0-95.7)	76.9 (54.0-99.8)
NPV	60.6 (54.5-66.6)	70.9 (64.3-77.4)
OPTICS		
Sensitivity	8.2 (3.1-13.3)	12.5 (4.3-20.6)
Specificity	96.2 (93.1-99.2)	97.0 (94.1-99.9)
PPV	60.0 (35.2-84.8)	66.7 (39.9-93.3)
NPV	59.8 (53.7-65.8)	69.9 (63.2-76.5)
H2FPEF		
Sensitivity	19.1 (11.7-26.4)	15.4 (6.6-21.2)
Specificity	90.4 (85.8-95.0)	89.5 (84.2-94.7)
PPV	58.3 (42.2-74.4)	41.7 (21.9-61.4)
NPV	61.3 (55.0-67.6)	68.4 (61.4-75.3)

The values in parentheses represent the 95% CIs.

NPV = negative predictive value; POISE = Prediction Of raISEd wedge pressures; PPV = positive predictive value.

In the current cohort, POISE had misdiagnosed 4 patients with postcapillary PH despite having a PCWP of 15 or below. Closer analysis of these 4 patients suggests these were patients with significant echocardiographic parameters of left heart dysfunction (mean E/e’ ratio 31.3 ± 12.4; ¾ dilated LA) with a PCWP just below cutoff (Supplemental Table 5).

### Missing data analysis

A summary of missing quantitative data is provided in Supplemental Table 5.

Formal assessment of missing quantitative data was conducted using Little’s MCAR test, which was significant (chi-square = 1,069.71; *df* = 764; *P* < 0.001), indicating the data were not missing completely at random. A comparison of covariates relevant to the clinical model between included (n = 266) and excluded participants (n = 291) identified additional systematic differences in LVEF, PASP, and summation of SV1 + RV6 on ECG (*P* < 0.001) (Supplemental Table 6).

To address potential bias from the missing data, sensitivity analysis was performed by adding SV1 + RV6 on ECG (mm), LVEF, and PASP as covariates to the model (Supplemental Table 7). In this adjusted analysis, obesity, E/e’, and LA dilatation remained significant independent predictors. The association for AF was attenuated (adj-OR: 2.061; 95% CI: 0.898-4.730; *P* = 0.088), suggesting that although AF remains a clinically relevant factor, its predictive value is partially accounted for by LVEF, PASP, and SV1 + RV6 on ECG.

## Discussion

In the present study, we developed and validated a novel clinical prediction tool (POISE) to aid in the prediction of postcapillary PH in an Asian population. With the use of 4 simple clinical variables, POISE was able to predict an elevated PCWP in 10% of patients at the risk of misdiagnosing <3% of patients. Despite its relatively limited sensitivity, it outperforms existing risk scores in our Asian cohort while maintaining a reasonably high specificity (Central Illustration).

**Central Illustration:**
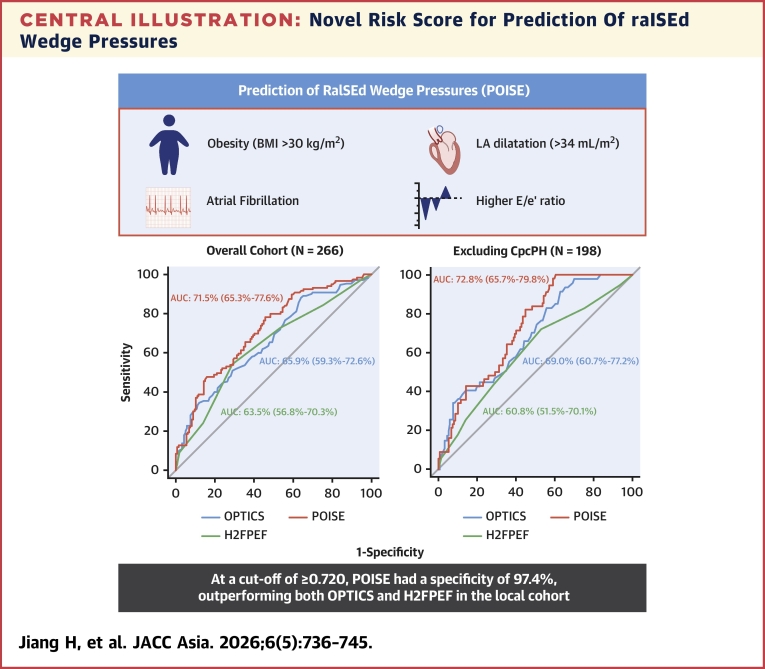
Novel Risk Score for Prediction Of raISEd Wedge Pressures Description of components of a novel risk score for POISE (Prediction Of raISEd wedge pressures), with comparisons to existing risk scores. POISE outperformed existing risk scores in terms of area under the curve (AUC) and specificity at prespecified cutoff values. BMI = body mass index; CpcPH = combined pre- and postcapillary pulmonary hypertension; LA = left atrial.

Despite validated results in Western cohorts with specificities of up to 100% and AUCs exceeding 0.8,^4^ the performance of the OPTICS and H2FPEF scores in our local population was not as promising. Both scores performed comparatively poorer with lower AUC in comparison with European cohorts (0.64-0.69 vs 0.79-0.81)^4^ and specificities of 97% for the OPTICS and 90% for the H2FPEF score. The poorer performance of both scores in our cohort compared with European cohorts may stem from several factors. There are known to be differences in PH characteristics between Asian and Western cohorts.^5^^,^^7^ Compared with European cohorts, the present cohort of patients with postcapillary PH had fewer risk factors for left heart dysfunction;^14^ the present cohort was younger in age (mean age 61.5 ± 14 vs 69 ± 11), with lower proportion of women (42% vs 59%), obesity (24% vs 46%), and AF (23% vs 42%). The lower prevalence of typical risk factors for left heart dysfunction, along with inherent differences in PH characteristics,^7^ may affect the applicability of these scores in an Asian cohort and make it inherently more difficult to predict postcapillary PH.

With a C-statistic of 0.72, sensitivity of 10%, and specificity of 97%, POISE outperformed both the OPTICS and H2FPEF scores in our local cohort. Nonetheless, we acknowledge the relatively lower sensitivity of POISE and lack of perfect specificity as limitations of the score. Close analysis of the 4 patients misdiagnosed by our risk score suggests they are at high risk of postcapillary PH, with significant LA dilatation and high E/e’ values and may very well develop postcapillary PH in the future. Furthermore, we evaluated POISE in the context of patients with CpcPH, which was not evaluated previously in the OPTICS and H2FPEF scores.^4^ The presence of CpcPH is associated with a poorer prognosis^1^^,^^15^ and may suggest a possible treatable precapillary component especially with a PVR of >5 WU.^1^ In the present study, 37% of the cohort was diagnosed with CpcPH on RHC. Excluding these patients, POISE maintained a good AUC of 0.749 with good specificity (98%).

Although RHC remains the gold standard for diagnosing and classifying PH,^1^ the limited uptake of RHC in parts of Asia remains a significant barrier to starting of treatment. This is for a variety of reasons including lack of expertise and resources and patient preference for avoidance of invasive procedures because of social and cultural norms.^16^ As such, a proportion of patients are diagnosed and started on treatment without RHC.^5^^,^^17^ In this setting, the use of POISE may aid in noninvasively identifying a subset of patients with high probability of postcapillary RHC, allowing for appropriate management of these patients and the prioritization of RHC to patients who may benefit most from invasive testing in resource-limited centers. Importantly, although the high specificity (>97%) of the score are aids in the identification of postcapillary PH, the scores are unable to rule out concomitant precapillary PH. In patients in whom clinical suspicion of a precapillary component is high, this does not negate the need for a RHC even if the risk score is positive.

A common pitfall of risk scores is the use of multiple variables or complex variables, which subsequently curtails the uptake.^18^^,^^19^ POISE consists of 4 variables, all of which are available in clinical practice. Of the 4 variables included in the novel risk score, 3 overlap with the OPTICS (obesity, AF, LA dilatation) and H2FPEF scores (obesity, AF, E/e’ ratio), respectively, underscoring their significance in the prediction of postcapillary PH. Interestingly, diabetes mellitus and dyslipidemia were both nonsignificant in the present cohort despite being known risk factors for postcapillary PH.^20^, ^21^, ^22^ In particular, echocardiographic parameters including LA volume index (suggesting chronically elevated LA pressures^23^) and E/e’ ratio (a predictor of left ventricle filling pressures^22^^,^^24^^,^^25^) appeared as important independent predictors of postcapillary PH despite exclusion of patients with reduced LVEF.^26^ The association between AF and raised PCWP was attenuated after adjusting for LVEF, PASP, and SV1 + RV6 (*P* = 0.088) as part of missing data analysis, even after exclusion of patients with LVEF <50%. This suggests that although AF remains a clinically relevant factor, other variables (obesity, E/e’ ratio, LA dilatation) were more robust. In future, the use of machine learning algorithms also holds promise to further improve risk stratification scores; a machine learning model was remarkably able to accurately diagnose 70% of patients with postcapillary PH while maintaining a specificity of 100% in a European cohort, although its routine use may be limited by its complexity.^27^

### Study strengths and limitations

To the best of the authors’ knowledge, this is the largest contemporary study to develop and validate a risk score for noninvasive prediction of postcapillary PH in an Asian cohort. The novel score uses 4 practically available variables, allowing for ease of clinical use and outperforms existing scores in an Asian cohort. Nonetheless, limitations exist. First, as a single-center retrospective review, this study comes with the inherent limitations associated with it, including missing data. There is also a risk of selection bias, although this may be partly mitigated as a large tertiary center receiving referrals for all cases of PH for consideration for RHC. Second, the development of the novel risk score for postcapillary PH included all patients who had undergone RHC regardless of indication, and the applicability to any specific subgroup may thus be reduced. Nonetheless, sensitivity analysis excluding patients with CpcPH demonstrated similar results. Third, the novel risk score was only validated via internal bootstrapping and may benefit further from future prospective validation in other external cohorts. Fourth, the score is unable to exclude concomitant precapillary PH and clinical judgment will still need to be exercised. Fifth, the current study only evaluated resting RHC and not exercise RHC, which may have helped unmask further cases of postcapillary PH. However, exercise RHC is not commonly available and there is no fixed consensus on the interpretation of exercise RHC given the complexity of physiological response.^28^^,^^29^ Hence, we chose to focus on resting RHC in the current study although the use of exercise RHC may be a topic for future study. Last, this study was performed in a single Asian country with predominantly Chinese ethnicity and smaller proportions of Malay and Indian ethnicity. Thus, the results may not be fully applicable to other Asian cohorts. This will need to be validated in other Asian populations.

## Conclusions

In a multiethnic Asian population, the high specificity and better performance (vis à vis existing scores) of POISE may help in accurately identifying a subset of patients with PH with postcapillary PH, potentially reducing the need of RHC in this group. The low sensitivity precludes its use as a routine screening tool, which is not the intent of this score. As with all risk scores, this is meant as an aid to the clinician and not to replace clinical judgment.

### Data Availability

The data that support the findings of this study are available from the corresponding author on reasonable request.

## Funding Support and Author Disclosures

Funding was not available for this study. Dr Yap has received speaker honoraria from Abbott, Biosensors, Biotronik, Boston Scientific, Edwards, GE Healthcare, J&J, Kaneka, Medtronic, and Terumo. Dr Low received consultancy fees and is on the advisory boards of Janssen and Boehringer Ingelheim and is on the steering committee and received research grants from Boehringer Ingelheim. All other authors have reported that they have no relationships relevant to the contents of this paper to disclose.
