# Supplementary material for: Development of a Noninvasive Prediction Score for Post-Capillary Pulmonary Hypertension in a Multi-Ethnic Asian Population
Source: JACC Asia. 2026 May 5;6(5):736–45. doi: 10.1016/j.jacasi.2026.03.012 (PMC13153881; doi:10.1016/j.jacasi.2026.03.012)
Supplement: Supplemental Tables 1-7 and Supplemental Figures 1-4 [file mmc1.docx]

**Supplemental Materials**

**Legend**

Supplemental Tables 1-2: Overview of OPTICS and H2FPEF scores

Supplemental Table 3: Decision curve analysis comparing net benefit of POISE with OPTICS and H2FPEF scores.

Supplemental Table 4: Overview of patients misdiagnosed by POISE

Supplemental Table 5. Overview of missing data used in MCAR analysis

Supplemental Table 6. Comparison of differences in model covariates of included vs excluded cases

Supplemental Table 7. Sensitivity Analysis of the Prediction Model Adjusted for Missingness Covariates

Supplemental Figure 1: CONSORT flow diagram

Supplemental Figure 2: Screenshot of online risk calculator for Prediction Of raISEd wedge pressures (POISE)

Supplemental Figure 3: Receiver Operator Curves of POISE, OPTICS, and H2FPEF in cohort of patients excluding patients with combined pre- and post-capillary pulmonary hypertension

Supplemental Figure 4. Internal validation via bootstrapping of POISE with removal of patients with combined pre- and post-capillary pulmonary hypertension

| **Clinical Variable** | **Values** | **Points** |
| --- | --- | --- |
| Obesity | Body Mass Index >30 kg/m^2^ | 22 |
| Diabetes Mellitus | Medical history of Diabetes Mellitus | 26 |
| Atrial Fibrillation | Paroxysmal or Persistent | 21 |
| Dyslipidemia | Non-fasting total cholesterol >5 mmol/l; HDL-C <1.0 mmol/l; LDL-C > 3 mmol/l | 17 |
| Valvular Surgery | Mitral or aortic valvular surgery without residual left valvular heart disease (less than mild on echocardiography) | 56 |
| SV1+RV6 on ECG | Sum of S wave in V1 and R wave in V6 on ECG (in mm) | 1x [SV1 + RV6] |
| Left atrial dilatation on echocardiography | Left atrial volume index > 34mL/m^2^ | 21 |

**Supplemental Table 1**. OPTICS risk score and point allocation of each parameter. A score of ≥104 was chosen as the cut-off for predicting post-capillary pulmonary hypertension

| **Clinical Variable** | **Values** | **Points** |
| --- | --- | --- |
| Obesity | Body Mass Index >30 kg/m^2^ | 2 |
| Hypertension | 2 or more hypertensive medication | 1 |
| Atrial Fibrillation | Paroxysmal or Persistent | 3 |
| Pulmonary Hypertension | Doppler Echocardiographic estimated Pulmonary Artery Systolic Pressure >35 mmhg | 1 |
| Elderly | Age >60 years | 1 |
| Filling Pressures | Doppler Echocardiographic E/e’ >9 | 1 |

**Supplemental Table 2**. H2FPEF risk score and point allocation of each parameter. A score of ≥6 was chosen as the cut-off for predicting post-capillary pulmonary hypertension

| **Model** | **C-Statistic (95% CI)** | **Net Benefit  (25% Threshold)** | **Net Benefit  (30% Threshold)** |
| --- | --- | --- | --- |
| **POISE** | **0.715 (0.653 – 0.777)** | **0.251 (0.180 - 0.321)** | **0.224 (0.154 - 0.294)** |
| **OPTICS** | 0.659 (0.593 – 0.726) | 0.227 (0.153 - 0.301) | 0.218 (0.149 - 0.286) |
| **H2FPEF** | 0.635 (0.568 – 0.703) | 0.198 (0.120 - 0.277) | 0.169 (0.092 - 0.247) |

**Supplemental Table 3.** Decision curve analysis comparing net benefit of POISE with OPTICS and H2FPEF scores.

| **Patient** | **Obesity** | **Atrial Fibrillation** | **LA dilatation** | **E/e’ ratio** | **Predicted score (cutoff >0.72)** | **Actual PAWP (mmHg)** |
| --- | --- | --- | --- | --- | --- | --- |
| A | Yes | No | Yes | 31.75 | 0.870 | 15 |
| B | No | Yes | Yes | 23.13 | 0.768 | 14 |
| C | No | Yes | Yes | 21.6 | 0.753 | 15 |
| D | No | No | No | 48.53 | 0.748 | 15 |

**Supplemental Table 4.** Analysis of patients misdiagnosed by POISE as post-capillary PH

| **Variable** | **Valid** | **Missing (n)** | **Missing (%)** |
| --- | --- | --- | --- |
| Height (m) | 266 | 0 | 0% |
| Weight (kg) | 266 | 0 | 0% |
| BMI | 266 | 0 | 0% |
| LA volume index | 266 | 0 | 0% |
| E/e’ ratio | 266 | 0 | 0% |
| Pulmonary artery systolic pressure | 247 | 19 | 7.1% |
| Left ventricle ejection fraction | 266 | 0 | 0% |
| NT-proBNP | 266 | 0 | 0% |
| Hemoglobin | 266 | 0 | 0% |
| Platelet | 266 | 0 | 0% |
| Creatinine | 266 | 0 | 0% |
| Sodium | 266 | 0 | 0% |
| Potassium | 266 | 0 | 0% |
| Heart Rate (BPM) | 246 | 20 | 7.5% |
| Mean right atrial pressure (mmHg) | 253 | 13 | 4.9% |
| Mean pulmonary artery pressure (mmHg) | 266 | 0 | 0% |
| Right ventricle systolic pressure (mmHg) | 258 | 8 | 3.0% |
| Left ventricle systolic pressure (mmHg) | 203 | 68 | 25.6% |
| Aortic systolic blood pressure (mmHg) | 232 | 34 | 12.8% |
| Aortic diastolic blood pressure (mmHg) | 232 | 34 | 12.8% |
| Cardiac output | 253 | 13 | 4.9% |
| Pulmonary vascular resistance | 259 | 7 | 2.6% |

**Supplemental Table 5.** Overview of missing data used in MCAR analysis

| Characteristic | Included (n = 266) | Excluded (n = 291) | p-value* |
| --- | --- | --- | --- |
| Age>60 | 159 (59.8%) | 191 (65.6%) | 0.088² |
| Body Mass Index >30 | 49 (18.4%) | 36 (12.4%) | **0.019**² |
| Smoker | 29 (10.9%) | 39 (13.4%) | 0.089² |
| Diabetes | 86 (32.3%) | 98 (33.7%) | 0.375² |
| Hypertension | 140 (52.6%) | 152 (52.2%) | 0.319² |
| Atrial Fibrillation | 38 (14.3%) | 132 (45.4%) | **<0.001**² |
| SV1 + RV6 on ECG | 12.0 (8.0, 18.0) | 15.0 (9.0, 25.0) | **<0.001**¹ |
| Left atrial volume index >34 cm^2^/m^2^ | 106 (39.8%) | 248 (85.2%) | **<0.001** |
| E/e’ Ratio | 10.0 (7.9, 14.1) | 16.2 (12.0, 23.3) | **<0.001**¹ |
| Left ventricular ejection fraction | 60.0 (56.0, 64.0) | 48.0 (27.0, 60.0) | **<0.001**¹ |
| Pulmonary artery systolic pressure | 52.0 (39.0, 65.0) | 44.0 (30.0, 56.0) | **<0.001**¹ |

¹ Calculated using **Mann-Whitney U test** for continuous variables.
² Calculated using **Pearson’s Chi-square test** for categorical variables.

**Supplemental Table 6.** Comparison of differences in model covariates of included vs excluded cases

|  | **Adjusted Odds Ratio**  **(95% CI)** | **P-value** |
| --- | --- | --- |
| Body Mass Index >30 | 2.713 (1.330, 5.534) | **0.006** |
| Atrial Fibrillation | 2.061 (0.898, 4.730) | 0.088 |
| Left atrial volume index >34 cm^2^/m^2^ | 2.011 (1.161, 3.809) | **0.032** |
| E/e’ Ratio | 1.059 (1.007, 1.114) | **0.026** |
| SV1 + RV6 on ECG* | 1.000 (0.967, 1.035) | 0.984 |
| Left ventricular ejection fraction* | 0.992 (0.941, 1.046) | 0.768 |
| Pulmonary artery systolic pressure* | 1.001 (0.987, 1.015) | 0.900 |

**Supplemental Table 7.** Sensitivity Analysis of the Prediction Model Adjusted for Missingness Covariates (as listed in Supplementary Table S6)


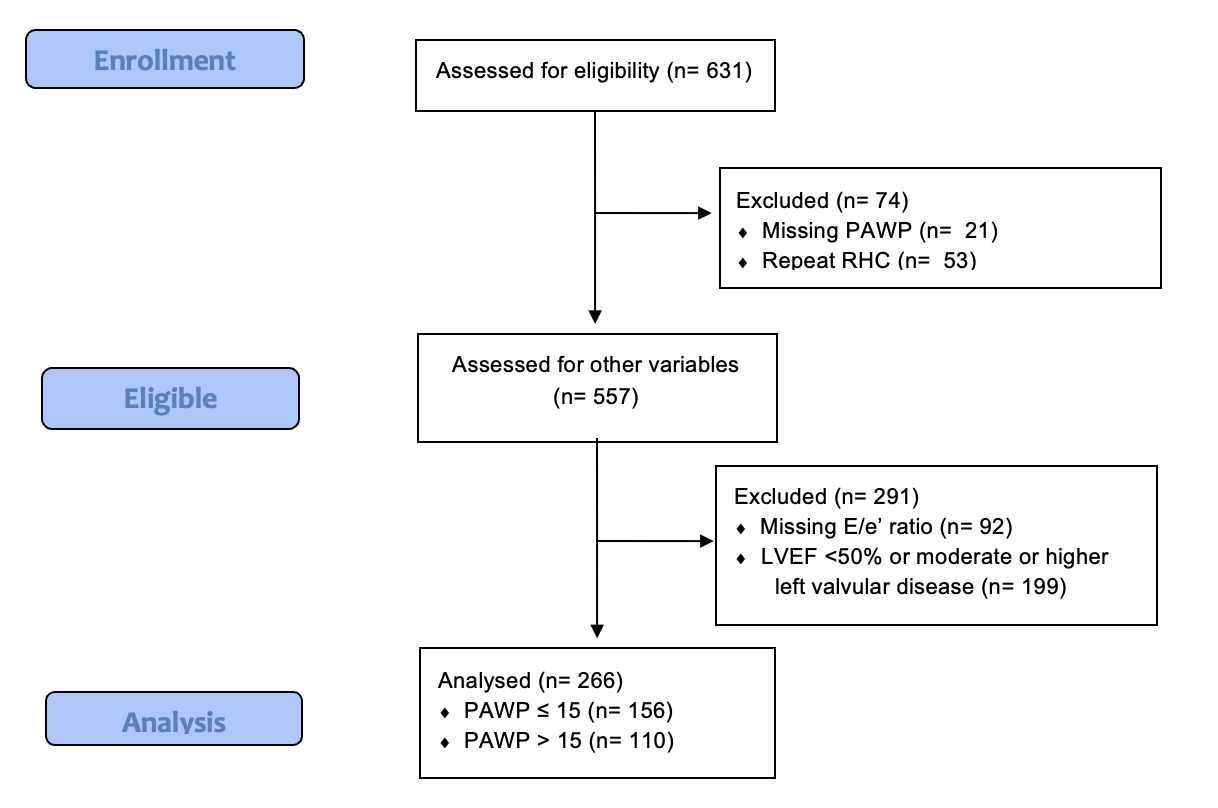


**Supplemental Figure 1.** Flow diagram depicting patient selection process


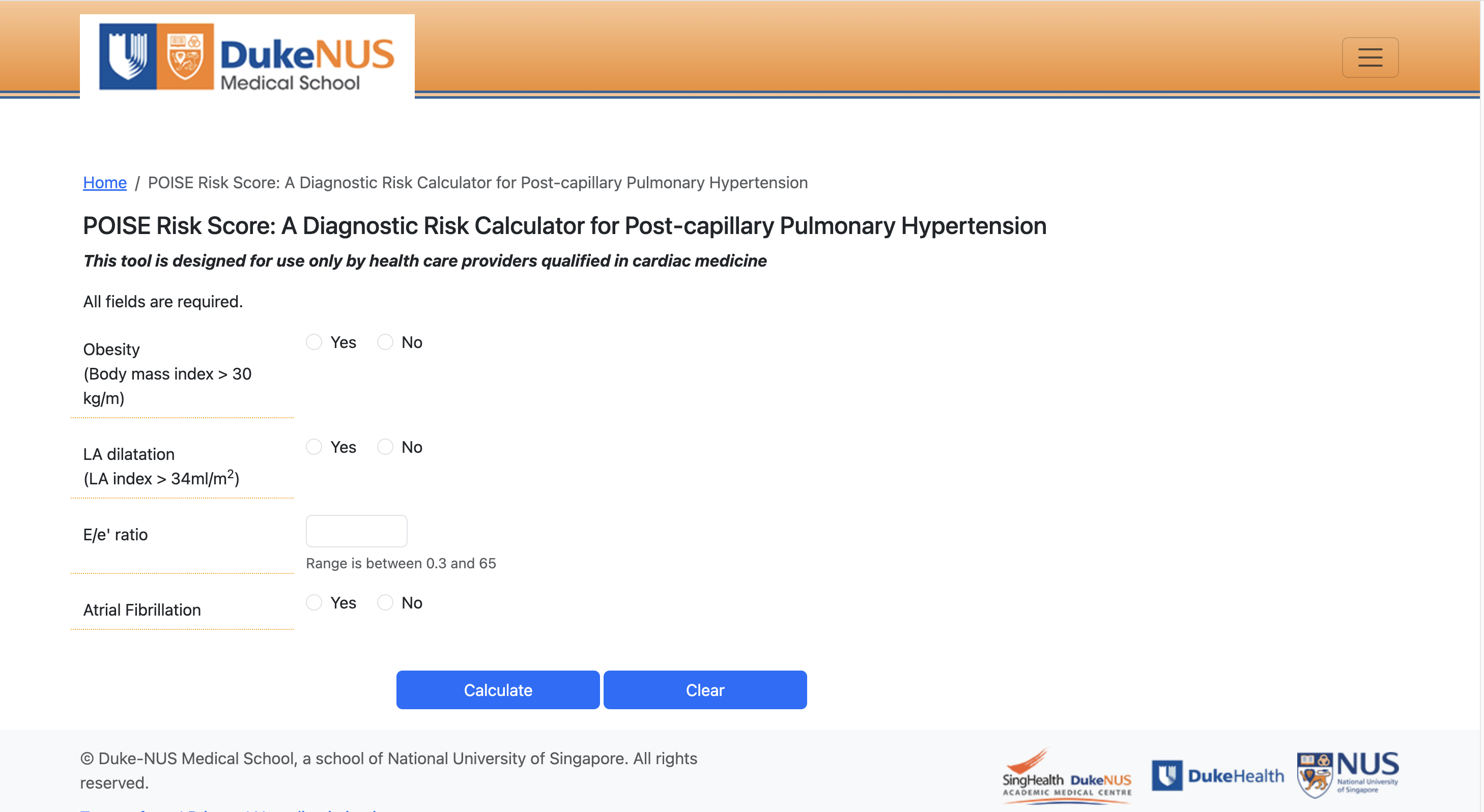


**Supplemental Figure 2**: Screenshot of online risk calculator for Prediction Of raISEd wedge pressures (POISE). Available from <https://webapps.duke-nus.edu.sg/tools/POISE>.


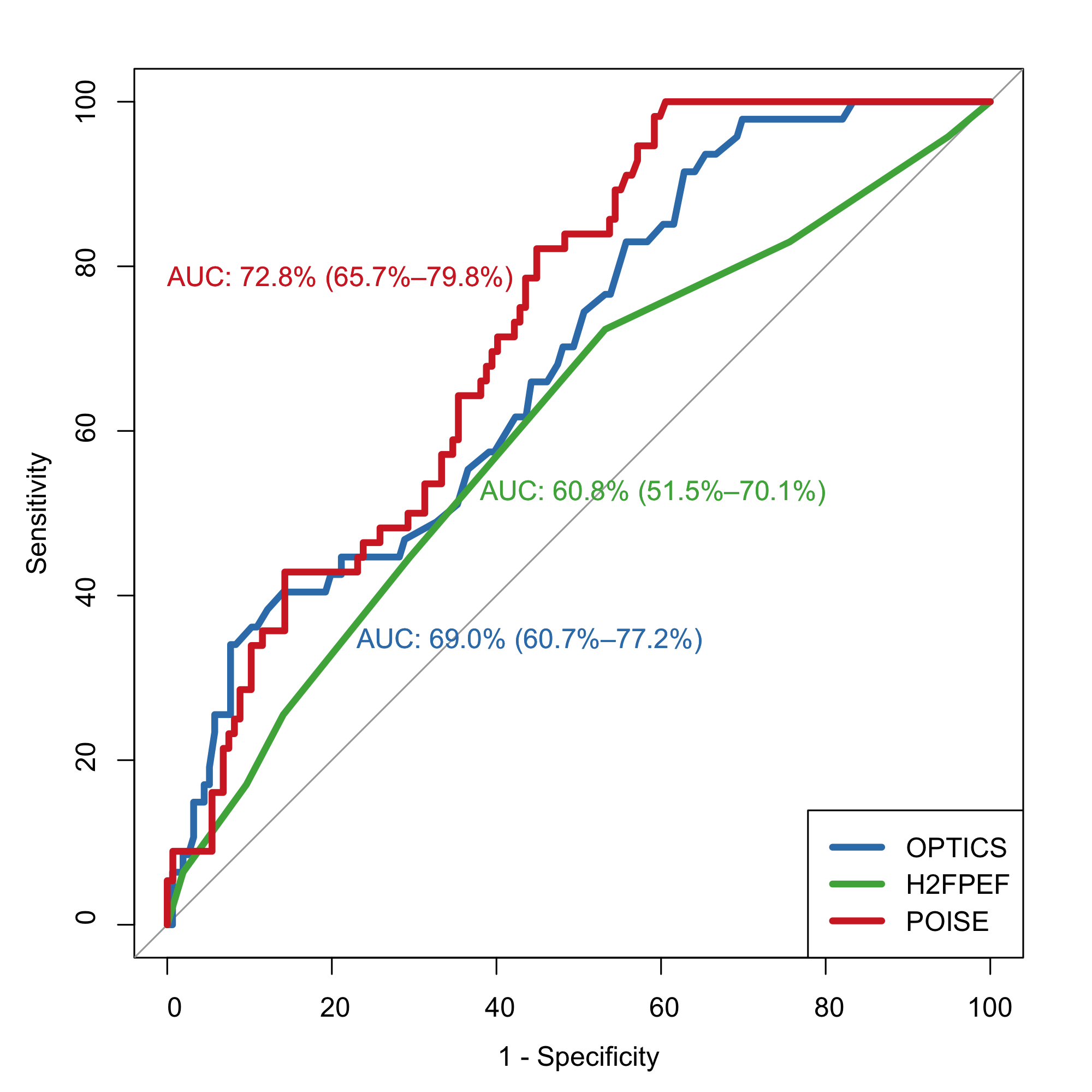


**Supplemental Figure 3.** Receiver Operator Curves of POISE, OPTICS, and H2FPEF in cohort of patients excluding patients with combined pre- and post-capillary pulmonary hypertension


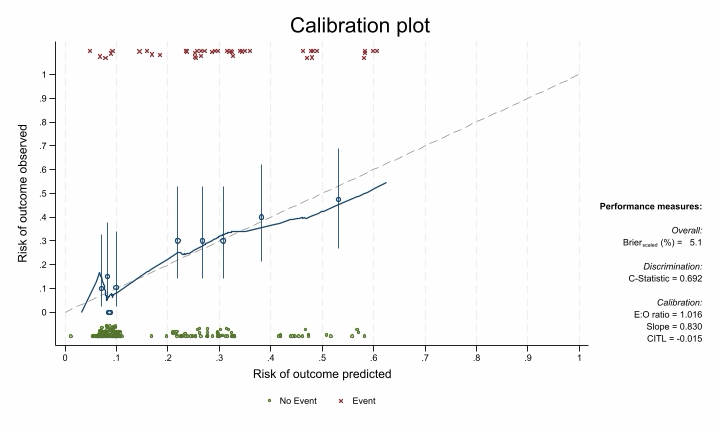


**Supplemental Figure 4.** Internal validation via bootstrapping of POISE with removal of patients with combined pre- and post-capillary pulmonary hypertension. C-statistic = 0.692 (95% CI 0.572 – 0.796).
